# Autoimmune glial fibrillary acidic protein astrocytopathy mimicking acute ischemic stroke: a case report

**DOI:** 10.3389/fimmu.2026.1768290

**Published:** 2026-04-22

**Authors:** Xianfeng Hou, Xun Mo, Ting Chen, Kaibin Qin, Shanshan Yu

**Affiliations:** 1 Department of Neurology, The Second People’s Hospital of Guiyang (Jinyang Hospital)/The Affiliated Jinyang Hospital of Guizhou Medical University, Guiyang, Guizhou, China; 2 Department of Neurology, Guiyang Public Health Clinical Center, Guiyang, Guizhou, China

**Keywords:** autoimmune GFAP astrocytopathy, case report, diffusion-weighted imaging, neuroimmunology, stroke mimic

## Abstract

Autoimmune glial fibrillary acidic protein (GFAP) astrocytopathy is a recently identified autoimmune disorder of the central nervous system. It commonly manifests as meningitis, encephalitis, myelitis, or optic neuritis. However, presentations resembling stroke are exceedingly rare. Here, we report a case of autoimmune GFAP astrocytopathy initially misdiagnosed as acute ischemic stroke. The correct diagnosis was later established through the detection of GFAP antibodies in both the patient’s serum and cerebrospinal fluid (CSF) using a cell-based assay (CBA). The patient initially responded to corticosteroid therapy; however, a relapse occurred during the tapering phase. After one year of treatment with oral prednisone and azathioprine, the patient achieved remission, with a subsequent negative result for GFAP antibodies. At the three-year follow-up, no recurrence was observed.

## 1 Introduction

Autoimmune glial fibrillary acidic protein (GFAP) astrocytopathy is a novel autoimmune disorder of the central nervous system first identified in 2016. GFAP-IgG in cerebrospinal fluid is a specific disease biomarker (1), although its pathogenesis remains incompletely understood. Many cases are preceded by a viral prodrome or associated with malignancy (2–5). Common clinical manifestations include encephalitis, meningoencephalitis, myelitis, optic neuritis, and psychiatric symptoms (4). Several less common presentations have been documented, such as area postrema syndrome (6), syndrome of inappropriate antidiuretic hormone secretion (7), intractable hiccups and nausea (8), and visual field defects (9). Magnetic resonance imaging (MRI) is central to diagnosis, with over 74% of patients showing T2-weighted or FLAIR hyperintensities in the brain and/or spinal cord, whereas diffusion-weighted imaging (DWI) sequences are typically normal in GFAP astrocytopathy. A characteristic radiologic feature is radial linear perivascular enhancement perpendicular to the lateral ventricles on contrast-enhanced MRI (2, 4, 5). Most patients respond well to corticosteroids, though refractory or relapsing disease may require intravenous immunoglobulin, plasma exchange, or other immunosuppressants such as rituximab, mycophenolate mofetil, or cyclophosphamide (6).

We describe a 20-year-old male initially misdiagnosed with acute ischemic stroke, in whom autoimmune GFAP astrocytopathy was later confirmed by detection of GFAP-IgG in both serum and CSF via cell-based assay. Treatment with corticosteroids and immunosuppressants led to sustained clinical improvement, with no relapse over a 3-year follow-up.

## 2 Case report

**Figure 1:**
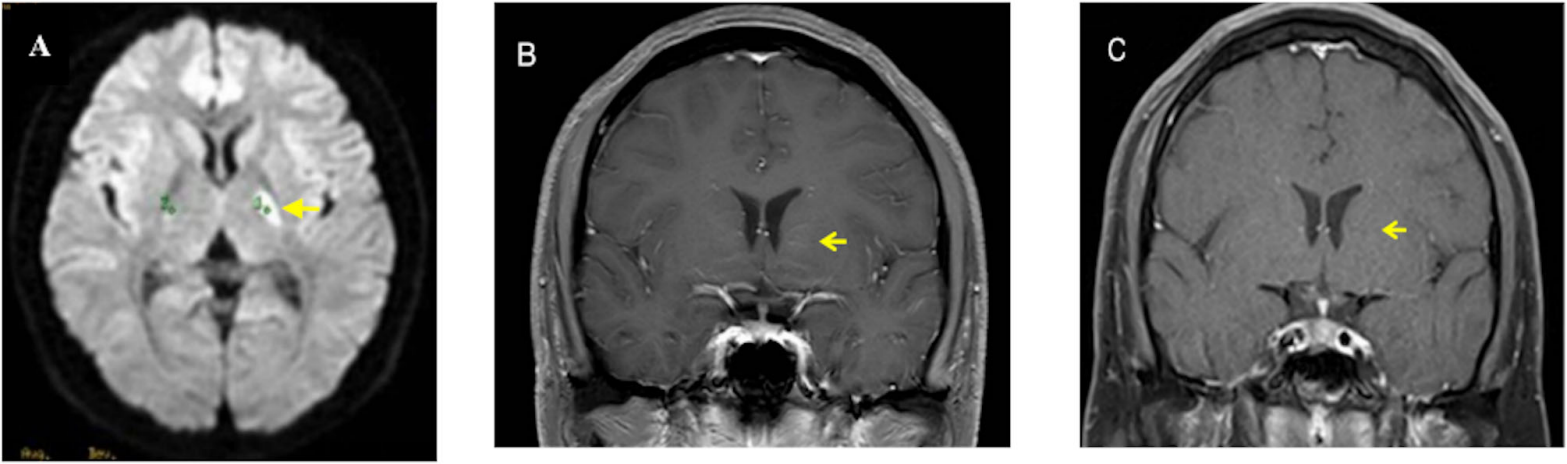
Brain MRI findings at different disease stages. **(A)** Axial diffusion-weighted image obtained during the first episode (June 2020) showing a hyperintense lesion in the left basal ganglia (arrow), initially misinterpreted as acute ischemic stroke. **(B)** Coronal contrast-enhanced T1-weighted image obtained during the second episode (August 2020) revealing characteristic radial linear perivascular enhancement perpendicular to the lateral ventricles (arrow), a hallmark of autoimmune GFAP astrocytopathy. **(C)** Coronal contrast-enhanced T1-weighted image obtained 3 months after immunosuppressive therapy (November 2020) demonstrating complete resolution of the previous periventricular enhancement.

**Figure 2:**
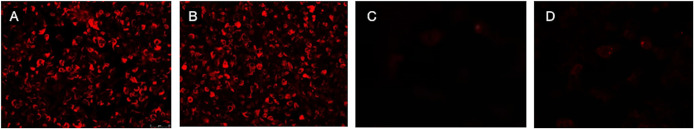
Cell-based assay results for GFAP-IgG at different time points. **(A, B)** Serum **(A)** and cerebrospinal fluid **(B)** samples obtained at initial diagnosis (August 2020) both tested positive for GFAP-IgG at a titer of 1:32. **(C)** CSF GFAP-IgG became negative at 3-year follow-up (November 2023). **(D)** Serum GFAP-IgG also became negative at the same time point, coinciding with sustained clinical remission.

A previously healthy 20-year-old male initially presented in June 2020 with sudden-onset right hemiparesis. Initial brain MRI demonstrated a focal area of restricted diffusion in the left basal ganglia on DWI (**Figure 1A**), while magnetic resonance angiography (MRA) revealed no vascular abnormalities. Contrast-enhanced sequences showed no corresponding abnormalities. The patient was managed as acute ischemic stroke and achieved complete functional recovery (modified Rankin Scale (mRS) score 0).Two months later, at the time of the second episode, he developed acute left hemiparesis and dysarthria. Repeat MRI revealed bilateral basal ganglia hyperintensities on T2-weighted imaging (T2WI) and DWI, with minimal apparent diffusion coefficient (ADC) reduction. Notably, these lesions did not conform to vascular territories. In addition, contrast-enhanced T1-weighted imaging demonstrated characteristic radial linear periventricular enhancement perpendicular to the ventricular walls (**Figure 1B**)—a finding not present on the initial MRI. Comprehensive evaluation detected GFAP-IgG antibodies in both serum and cerebrospinal fluid (CSF) via cell-based assay (titer 1:32 in both compartments, **Figures 2A, B**), confirming autoimmune GFAP astrocytopathy. Intravenous methylprednisolone pulse therapy (1000 mg/day for 5 days followed by gradual tapering) resulted in complete neurological recovery. Three-month follow-up MRI demonstrated resolution of periventricular enhancement (**Figure 1C**); antibody testing at that time remained positive. Subsequent steroid tapering was followed by clinical relapse characterized by right limb dysesthesia, accompanied by elevated CSF pressure (250 mmH_2_O). Repeat antibody testing showed rising serum titer (1:100) and CSF titer remained 1:32. Mycophenolate mofetil (0.5 g twice daily) with low-dose steroids achieved sustained remission. At three-year follow-up, the patient remained recurrence-free, with negative GFAP-IgG in both serum and CSF (**Figures 2C, D**).

## 3 Discussion

To our knowledge, a stroke-like presentation mimicking acute ischemic stroke has not been previously described in autoimmune GFAP astrocytopathy. Given the unequivocal DWI restriction during the first episode—a finding that typically triggers acute stroke management—the initial misdiagnosis was understandable. However, the patient experienced rapid and complete recovery after the first episode, followed by contralateral relapse months later. Imaging revealed bilateral non-vascular territory lesions, absence of the initial lesion on follow-up FLAIR, and characteristic perivascular enhancement. These atypical features ultimately guided the correct diagnosis. Notably, DWI restriction is rare in GFAP astrocytopathy, occurring in only approximately 3% of cases (8), and the patient had no clear history of prodromal infection, factors that further increased diagnostic difficulty.

In young patients presenting with acute neurological deficits and DWI hyperintensity, the differential diagnosis should not be limited to acute ischemic stroke. Understanding the pathophysiological mechanisms underlying DWI hyperintensity is crucial: DWI hyperintensity reflects restricted water diffusion, most commonly due to cytotoxic edema—which can arise from ischemic stroke (energy failure) or severe neuroinflammation (glial cell activation leading to cellular swelling) (10). In addition, some hypercellular tumors (e.g., lymphoma) and infectious lesions (e.g., brain abscess) may also show DWI hyperintensity due to high cellular density, and metabolic diseases (e.g., mitochondrial disorders, hepatic encephalopathy, uremic encephalopathy) can cause cytotoxic edema with DWI hyperintensity (14, 15).

Although ischemic stroke is the most common cause of DWI hyperintensity, the nature of the first episode in our patient remains uncertain. The initial DWI lesion met the imaging criteria for acute ischemic stroke, and the patient’s rapid recovery was consistent with a mild ischemic insult. However, bilateral basal ganglia involvement did not conform to a single vascular territory, and head and neck MRA along with cardioembolic screening showed no abnormalities. Importantly, follow-up FLAIR three months after the first episode revealed no residual gliotic changes in the region of the initial DWI lesion, whereas true ischemic infarction typically evolves into focal encephalomalacia or gliosis. Thus, while a small-vessel ischemic event cannot be entirely excluded, the overall evidence favors an inflammatory etiology. Furthermore, the patient’s rapid recovery followed by contralateral relapse two months later is uncommon in ischemic stroke. The appearance of bilateral non-vascular territory DWI lesions and characteristic radial perivascular enhancement during the second episode—a hallmark imaging feature of GFAP astrocytopathy (2, 4, 5)—further supported an inflammatory etiology.

In addition, we systematically excluded other potential etiologies:

- Primary CNS vasculitis was considered unlikely due to repeatedly negative vasculitis antibody panels, absence of multifocal infarcts, and normal MRA without segmental vascular stenosis.
- Autoimmune encephalitis (e.g., anti-NMDAR encephalitis) can involve the basal ganglia (11, 12), but comprehensive neuronal antibody testing (including MOG-IgG, MBP-IgG, AQP4-IgG, NMDAR-IgG, IgLON5-IgG, DPPX-IgG, GlyR-IgG, DRD2-IgG, GAD65-IgG, mGluR5-IgG, mGluR1-IgG, AMPA1-IgG, AMPA2-IgG, LGI1-IgG, CASPR2-IgG, GABABR-IgG) was negative in both serum and CSF, and oligoclonal bands were also absent.
- Infectious encephalitis (e.g., viral encephalitis) can involve the basal ganglia and present with DWI hyperintensity (13), but the patient had no history of prodromal infection, and CSF metagenomic next-generation sequencing (mNGS) as well as serological tests were negative. Metabolic diseases (e.g., hepatic encephalopathy, uremic encephalopathy) may present with DWI hyperintensity (14, 15) but are typically accompanied by other systemic involvement or family history, for which there was no evidence in this case.
- Neoplastic diseases (e.g., primary CNS lymphoma) can show DWI hyperintensity (16), but chest, abdominal, and urogenital CT, tumor markers, and onconeural antibody panels were all unremarkable.
- Infectious space-occupying lesions (e.g., brain abscess) may also show DWI hyperintensity due to viscous pus (17), but there were no signs of infection and imaging features were not supportive.

Beyond MRI, the detection of GFAP antibodies in both serum and CSF was essential for confirming the diagnosis of autoimmune GFAP astrocytopathy. Although GFAP antibodies are not directly pathogenic, they reflect astrocytic inflammation likely mediated by GFAP-specific CD8+ T cells, microglia, and cytokines (1, 2). Consequently, autoimmune GFAP astrocytopathy is frequently associated with malignancies (e.g., teratomas, gliomas) and prodromal viral infections (6, 7, 9). Notably, our patient presented with neither of these conditions (negative chest/abdominal/urogenital CT, tumor markers, and onconeural antibodies), further compounding the diagnostic challenge. This underscores the importance of considering autoimmune GFAP astrocytopathy in clinical practice even in the absence of associated malignancies or viral infections.

Our patient responded initially to corticosteroids but relapsed during taper. Addition of mycophenolate mofetil achieved sustained remission, and at three-year follow-up, GFAP antibodies became negative in both serum and CSF. This is consistent with previous reports (1, 2, 4) and supports long-term immunosuppression as a cornerstone of therapy for this disease.

Although autoimmune GFAP astrocytopathy classically presents as meningoencephalomyelitis (1–4), recent studies have revealed a broader clinical spectrum, including isolated or atypical manifestations. The present case further expands the clinical spectrum of this disease, demonstrating that GFAP astrocytopathy can rarely present as a stroke mimic with isolated motor symptoms, without overt signs of encephalitis or meningitis. This presentation is anatomically plausible, as bilateral basal ganglia involvement typically manifests as motor dysfunction. Notably, elevated CSF pressure (250 mmH_2_O) during relapse supported an underlying inflammatory process. Despite the absence of typical encephalitic features, the characteristic MRI findings and repeatedly positive GFAP-IgG on cell-based assays were sufficient to establish the diagnosis. This atypical presentation underscores the importance of considering GFAP astrocytopathy in the differential diagnosis of stroke mimics, particularly when imaging reveals non-vascular territory lesions and periventricular enhancement.

## 4 Conclusion

Autoimmune GFAP astrocytopathy is a rare but treatable disorder with diverse manifestations. This case emphasizes the importance of considering stroke mimics and recognizing that DWI restriction does not invariably indicate ischemic infarction. Careful assessment of discordant features—such as fluctuating symptoms, non-vascular territory lesions, and specific MRI findings like perivascular enhancement—should prompt timely antibody testing. As shown here, early diagnosis followed by appropriate immunosuppressive therapy can lead to favorable long-term outcomes.

## Data Availability

The datasets presented in this article are not readily available because of ethical and privacy restrictions. Requests to access the datasets should be directed to the corresponding author.
